# The impact of bone histomorphometry, bone-related biomarkers, and FRAX^®^ on fractures patients with predialysis CKD

**DOI:** 10.1590/2175-8239-JBN-2024-0276en

**Published:** 2025-12-01

**Authors:** Mariana Fernandes Diz Lopes, Bernardo José Cardoso Fernandes, Maria Teresa Almeida e Sousa Martins da Rocha, Maria Lúcia Carvalho Costa, João Miguel Machado Dória Frazão, Ricardo de Morais Pereira

**Affiliations:** 1 Unidade Local de Saúde de São João, Departamento de Reumatologia, Porto, Portugal.; 2 Universidade de Porto, Faculdade de Medicina, RISE-Health, Departamento de Medicina, Porto, Portugal.; 3 Unidade Local de Saúde de São João, Departamento de Nefrologia, Porto, Portugal.

**Keywords:** Chronic Kidney Disease-Mineral and Bone Disorder, Fractures, Osteoporotic Fractures, Osteoporosis, Histomorphometry, Bone Remodeling, Bone Bank

## Abstract

**Introduction:**

Chronic kidney disease (CKD) patients are at increased risk of fracture. Whether the type of renal osteodystrophy (ROD) contributes to fracture risk is not currently established since bone biopsies are not frequently performed in clinical practice. We aimed to evaluate the association of ROD subtypes, bone biomarkers, and fracture risk assessed with the FRAX^®^ tool with the occurrence of fractures in predialysis CKD patients.

**Methods:**

Retrospective study with patients followed between 2014–2023. Blood tests, including bone-related biomarkers (BRB), and bone biopsies were performed at the beginning of follow-up. Data from dual x-ray absorptiometry (DXA) scan and clinically evident fractures were obtained from medical registries. Radiographs of the thoracic/lumbar spine were evaluated to detect vertebral fractures, and the FRAX^®^ index without bone mineral density (BMD) was calculated with the web-based tool.

**Results:**

Median follow-up time was 7.5 ± 3 years and 9.3% of the patients had a bone fracture, with an incidence of 12/1000 patient-year. Patients who had a fracture had higher phosphorus levels (4.1 mg/dL vs 3.5 mg/dL, p = 0.047). Histomorphometric subtypes and BRB were not associated with incidence of fractures nor with fracture risk assessed by FRAX^®^. There was a tendency for lower bone volume in the group with fractures (p = 0.057). FRAX^®^ (without BMD), regardless of the inclusion of CKD as secondary osteoporosis, showed an overall good diagnostic accuracy for predicting fractures in predialysis CKD.

**Conclusion:**

ROD subtypes were not associated with incidence of fractures in these patients. The discriminative ability of FRAX in this population emphasizes its usefulness in CKD predialysis patients.

## Introduction

Osteoporosis is a common skeletal disorder that affects bone macro and microarchitecture and bone mineral density (BMD), resulting in bone fragility and fragility fractures^1^. All fractures are a major burden, since they are associated with high morbidity and mortality^1^. Chronic kidney disease (CKD) patients have a complex physiopathology of bone fragility given the development of mineral bone disorder (CKD-MBD) and are at increased fracture risk^2^. CKD-MBD includes metabolic disorders of calcium, phosphorus, parathyroid hormone, and fibroblast growth factor (FGF)-23 that lead to disruption of bone morphology (renal osteodystrophy), vascular calcification, and cardiovascular death^3^. Bone turnover, mineralization, and volume (TMV) classification, proposed by Kidney Disease: Improving Global Outcomes (KDIGO), are used to establish the different subtypes of renal osteodystrophy (ROD), which is assessed through bone biopsies and histomorphometric analysis^4^.

In patients with CKD grade 1–3, the risk of osteoporosis and fracture are similar to that of the general population^5,6^. However, challenges emerge when dealing with patients with CKD grade 4-5^5,6^. Dual x-ray absorptiometry (DXA) is the gold-standard for BMD and fracture risk assessment in clinical practice^7^. However, DXA does not discriminate between cortical and trabecular bone, does not distinguish between bone volume and bone mineralization conditions, and can be influenced by artifacts, such as ectopic calcifications or pathologic bone formation^2,5,6^. Therefore, the accuracy of the test may be compromised in some specific populations, such as CKD patients.

The value of bone circulating biomarkers in fracture risk is not established^8^. Several molecules are relevant in bone and mineral metabolism. Parathyroid hormone (PTH) has a central role in bone remodeling and in the Wnt/beta-catenin pathway; bone alkaline phosphatase (BALP) and total alkaline phosphatase (ALP) are markers of bone forming activity; fibroblast growth factor 23 (FGF-23) is a pleiotropic hormone that inhibits phosphate reabsorption and calcitriol production; sclerostin and dickkopf-related protein 1 (Dkk-1) are antagonists of osteoblastogenesis and indirectly affect receptor activator for nuclear factor kB ligand (RANKL), that binds to RANK receptor on osteoclasts and stimulates bone resorption and osteoprotegerin, blocking the interaction between RANK and RANKL^9^.

Fracture Risk Assessment (FRAX^®^) is a web-based tool available worldwide used to predict 10-year fracture risk^10^. Although it is used in CKD patients, it can underestimate fracture risk. Therefore, some adjustments are needed to improve its accuracy^11^.

Predicting fracture risk in CKD patients is still a challenge in clinical practice. Bone strength is determined not only by bone quantity (evaluated mostly with BMD assessed by DXA), but also by bone quality, determined by the microarchitecture and mechanical properties of the bone^12^. Whether ROD subtype and bone turnover, mineralization, and volume (TMV) classification parameters contribute to the assessment of fracture occurrence and fracture risk is not currently known. A large series study from Brazil and Uruguay was not able to find an association between ROD and fracture incidence^5,13^. As bone biopsies are not routinely performed given their invasive character and the need for specific equipment and training for histomorphometric analysis^14^, the use of non-invasive tools such as FRAX should be expanded.

The aims of this study were (i) to evaluate the incidence of fractures in a CKD predialysis cohort, (ii) to study the association between ROD subtypes and circulating bone biomarkers with fractures, and (iii) to evaluate the predictive value of the FRAX index for major osteoporotic fractures in this population.

## Methods

### Study Design and Population

This study was a retrospective evaluation of adult patients followed in our institution between 2014–2023 with a bone biopsy performed during the predialysis period, previously enrolled in a cross-sectional study by Pereira et al^8^. Patients were eligible if they had CKD stage 3 and 4 at the time of bone biopsy, were capable of giving informed consent, and had > 18 years of age. Exclusion criteria were a history of treatment with biphosphonates, denosumab or osteoanabolics (teriparatide) and previous parathyroidectomy. All patients signed an informed consent form. The study protocol was approved by the institutional ethics committee.

### Laboratory Tests

All patients had blood samples collected at the time of bone biopsy. Serum calcium, phosphorus, albumin, creatinine, and alkaline phosphatase were measured using an Olympus AU5400 analyzer (Olympus America, Centre Valley, PA, USA). Serum iPTH and 25-hydroxyvitamin D [25(OH)D] levels were assessed through an electrochemiluminescence immunoassay with a Cobas E411 analyzer (Roche Diagnostics, Mannheim, Germany). Bioactive sclerostin and free soluble RANKL (sRANKL) were measured in plasma samples by an enzyme immunoassay (Biomedica Medizinprodukte GmbH, Wien, Austria), according to the manufacturer’s instructions (detection range of 1.9–320 pmol/L and 0.2–40 pg/mL, respectively). Osteoprotegerin, DKK1, and intact FGF-23 were measured in plasma samples by a multiplex assay (Magnetic Luminex Assay, R&D Systems Inc., Minneapolis, MN, USA), according to the manufacturer’s protocol (detection range of 75–18,220 pg/mL, 202–49,060 pg/mL, and 11.8–2870 pg/mL, respectively). CKD stage was graded according to the CKD Epidemiology Collaboration equation.

### Bone Biopsy and Histomorphometry

All patients were submitted to a transiliac bone biopsy with a modified Bordier trephine at the beginning of follow-up. Bone biopsy was performed after a course of double-labelling tetracycline (doxycycline 200 mg twice daily for 3 days, which was repeated after 12 days; the biopsy was performed 3 days after the second labelling). Biopsy specimens were 5 mm in diameter and 10 mm in length. The specimen was dehydrated in alcohol, cleared with xylene, and embedded in methyl methacrylate. Undecalcified 5-μm sections were cut and stained with modified Masson-Goldner trichrome for static histomorphometric evaluation. Unstained 10-μm sections were prepared for fluorescent micro­scopy analysis of dynamic parameters. Bone histomor­phometry was performed with OsteoMeasure software (OsteoMetrics, Decatur, GA, USA). Static and dynamic parameters were examined according to the standards established by the American Society of Bone and Mineral Research^15^.

Following the KDIGO guidelines^4^, ROD was categorized according to the TMV classification into osteomalacia (low turnover, abnormal mineralization), adynamic bone (low turnover, normal mineralization), mixed uremic osteodystrophy (high turnover, abnormal mineralization), and hyperparathyroid-related bone disease (high turnover, normal mineralization).

Low bone turnover was defined as bone formation rate/bone surface (BFR/BS) <10.95 mm^3^/mm^2^/year and high bone turnover as BFR/BS >37.5 mm^3^/mm^2^/year. Bone volume was considered normal if bone volume/tissue volume (BV/TV) >16%. Mineralization was considered normal if mineralization lag time (Mlt) was <50 days. Biopsies were evaluated by two different observers.

### Fractures and Fracture Risk Evaluation

A fragility fracture was defined as a fracture resulting from a trauma equivalent to or less than a fall from one’s own height. Vertebral and nonvertebral fragility fractures were included. Medical registries were evaluated for the occurrence of clinical (symptomatic) fractures. Lateral radiographies of the spine from the last two years were available in 51 patients, which were reviewed by a rheumatologist for the detection of vertebral asymptomatic fractures, using the Genant semiquantitative method.

All information on risk factors for bone fractures was obtained from the medical history and included age, sex, weight and height, previous fractures, family history of hip fractures, smoking, alcohol intake (>3 U/day), rheumatoid arthritis, current or previous use of systemic corticosteroids for >3 months at a dose >5 mg/day and diagnosis of a disease convening secondary osteoporosis (chronic hepatic disease, type 1 diabetes mellitus, early menopause (<45 years), osteogenesis imperfecta, untreated hyperthyroidism, hypogonadism).

The 10-year risk of major bone and hip fracture was calculated with the Portuguese version of the FRAX calculator (https://frax.shef.ac.uk/FRAX/tool.aspx?country=53). High fracture risk according to the FRAX index without BMD, also considered as the cut-off for therapeutic intervention, was defined as ≥11% for major fractures and ≥3% for hip fractures^7^.

DXA data were collected when available. BMD at the femoral neck, total hip, and lumbar spine (L1–L4) was evaluated with the QDR Hologic Delphi machine.

### Statistical Analysis

Statistical analysis was performed using the SPSS version 29.0 (IBM SPSS Statistics, Chicago, IL, USA). Continuous data were expressed as mean and standard deviation (SD) or median and interquartile range (IQR), according to variable distribution. Categorical variables were expressed as frequencies and percentages.

The groups with and without fractures were compared, and p-values were calculated using Mann-Whitney U and Kruskal Wallis tests for continuous variables and chi-square and Fisher exact test for categorical variables.

To determine the ability of the FRAX tool to predict fractures, we constructed receiver operating characteristic (ROC) curves for each predictor variable: FRAX without BMD and FRAX without BMD considering CKD as secondary osteoporosis. We evaluated the area under the ROC curves (AUC) for each FRAX model. An AUC was considered good if >0.7 and excellent if >0.8. Sensitivity, specificity, and positive and negative predictive values were calculated. Values of p < 0.05 were considered statistically significant.

## Results

### Clinical, Demographic and Laboratory Characteristics According to Fracture Status

A total of 54 patients, mostly male (n = 43, 79.6%) and with a mean age of 72.2 ± 10.2 years were included. Median follow-up time was 7.5 ± 3 years. Two patients (3.7%) had clinically evident fractures (one hip fracture and one peroneal fracture) and asymptomatic vertebral fractures were identified in radiographies of 3 (5.9%) patients. This represents a fracture incidence of 12/1000 patient-year, with 9.3% of the patients experiencing a fracture.

Clinical, demographic, and biochemical charac­teristics in the two groups are summarized in Table 1. Mean age at the time of bone biopsy was 71.84 ± 4.4 years in the fracture group and 64. 7 ± 10 years in the no-fracture group. Most patients were male in both groups (n = 3, 60% and n = 40, 81.6%).

**Table 1:** Clinical, demographic and laboratory characteristics according to fracture status

	Patients with fractures during follow-up n = 5 (9.3%)	Patients without fractures during follow-up n = 49 (90.7%)	p-Value
Age (years) at the time of bone biopsy, mean (SD)	71.8 (4.4)	64.7 (10)	0.126
Age (years) at the end of follow-up, mean (SD)	79 (5.4)	71.5 (10.4)	0.117
CKD stage, n (%)			0.358
Stage 3	1 (20)	24 (49)	
Stage 4	4 (80)	25 (51)	
Gender			0.266
Female, n (%)	2 (40)	9 (18.4)	
Male, n (%)	3 (60)	40 (81.6)	
Chronic kidney disease etiology, n (%)			0.732
Hypertension	3 (60)	13 (26.5)	
Diabetes mellitus	2 (40)	13 (26.5)	
Chronic glomerulonephritis	0	6 (12.2)	
Chronic tubulointerstitial nephritis	0	8 (16.3)	
Autosomal dominant polycystic kidney disease	0	2 (4.1)	
Ischemic	0	1 (2)	
Indeterminate	0	6 (12.2)	
Current smoker, n (%)	0	1 (1.9)	0.747
Alcohol consumption, n (%)	0	4 (8.2)	0.670
Weight, median (IQR)	79 (16)	76 (16.5)	0.900
Height, mean (SD)	158.4 (6.7)	163 (7.9)	0.215
Body mass index, median (IQR)	32.8 (8.4)	28.1 (5.4)	0.366
Biochemistry, median (IQR)			
Creatinine, mg/dL	2.2 (1.1)	2.2 (0.5)	0.415
GFR, mL/min/1.73 m^2^	26.5 (15)	30.5 (12)	0.157
Hemoglobin, g/dL	11.5 (0.5)*	13.3 (1.9)*	0.008
Albumin, g/dL	4.08 (0.14)	4.2 (0.4)	0.848
Calcium, mg/dL	4.7 (0)	4.8 (0.4)	0.685
Phosphorus, mg/dL	4.1 (0.8)*	3.5 (0.9)*	0.047
25(OH) Vitamin D, ng/mL	12.5 (4.8)	16 (12.3)	0.221
Magnesium, mEq/L	1.8 (0.3)	1.6 (0.3)	0.957
C-reactive protein, mg/L	3.9 (4.4)	1.5 (3.5)	0.662
25(OH)D, ng/mL	13.5 (3)	16 (9.8)	0.221
ALP, U/L	75.5 (19)	71 (43)	0.815
iPTH, pg/mL	105.7 (40.6)	79.9 (93.7)	0.728
FGF-23, pg/mL	30.9 (10.8)	22.1 (21.1)	0.874
Sclerostin, pmol/L	83.9 (105)	58.4 (44.7)	0.986
DKK1, pg/mL	1004.5 (283.9)	738 (498.9)	0.366
sRANKL, pg/mL	2.7 (0.3)	2.5 (1.4)	0.231
Osteoprotegerin, pg/mL	1554.4 (408.3)	1404.1 (545.3)	0.385
Osteoporosis according to DXA, n (%)	0 (0)	5 (9.3)	0.665
Progression to kidney replacement therapy during follow-up, n (%)			1.000
Hemodialysis	2 (40)	18 (36.7)	
Peritoneal dialysis	0	2 (4)	
Death during follow-up, n (%)	1 (20)	4 (8.2)	0.397

Abbreviations – SD: standard deviation; CKD: chronic kidney disease; IQR: interquartile range; GFR: glomerular filtration rate; ALP: alkaline phosphatase; iPTH: intact parathyroid hormone; FGF-23: fibroblast growth factor 23; DKK1: dickkopf-related protein 1; RANKL: soluble receptor activator for nuclear factor kB ligand.

Hypertension (n = 3, 60%) and diabetes (n = 2, 40%) were the CKD etiologies in all the patients in the fracture group. The majority of patients had CKD stage 4 (n = 29, 53.7%) and during follow-up, 20 patients progressed to hemodialysis, 2 progressed to peritoneal dialysis, and 5 died (9.3%). There were no statistically significant differences between the two groups regarding patient progression to kidney replacement therapy.

Five patients (9.3%) had osteoporosis according to the T score from DXA, and none of these patients experienced a fracture during follow-up.

Mean serum calcium and phosphorus at the time of bone biopsy were, respectively, 9.4 ± 0.7 mg/dL and 3.5 ± 0.7 mg/dL. Median iPTH serum levels were 90.2 (85.4) pg/mL. Patients who had fracture had higher phosphorus levels (4.1 mg/dL vs 3.5 mg/dL, p = 0.047) and lower hemoglobin levels (11.5 mg/dL vs 13.3 mg/dL, p = 0.008).

There was a tendency for higher ALP and iPTH in the group with fractures, but these differences were non-significance. The remaining circulating bone biomarkers (FGF-23, sclerostin, DKK1, sRANKL and osteoprotegerin) were not associated with incidence of fractures.

### Bone Histomorphometry Analysis and Fractures

In the histomorphometric analysis, 40.7% (n = 22) of the patients had normal bone histology, 37% (n = 20) had low bone turnover with normal mineralization (adynamic bone disease), 20.4% (n = 11) had high bone turnover with normal mineralization (hyperparathyroid bone disease), and 1 patient had mixed uremic osteodystrophy (high bone turnover with abnormal mineralization). Cases of low bone turnover with abnormal mineralization (osteomalacia) were detected. Histomorphometric measurements and bone histology in relation to occurrence of fractures is described in Table 2. There was no association between histomorphometric subtypes and occurrence of fractures.

**Table 2:** Bone histology and histomorphometric parameters according to fracture status

	Patients with fractures during follow-up n = 5 (9.3%)	Patients without fractures during follow-up n = 49 (90.7%)	p-Value
Bone histomorphometric results, n (%)			0.847
Normal bone	3 (60)	19 (38.8)	
Adynamic bone disease	1 (20)	19 (38.8)	
Hyperparathyroid bone disease	1 (20)	10 (20.4)	
Mixed uremic osteodystrophy	0 (0)	1 (2)	
BFR/BS, mm^3^/mm^2^/year, median (IQR)	15.5 (5.2–20.4)	15.5 (8.9–38.0)	0.303
Turnover, n (%)			0.833
Normal	3 (60)	19 (38.8)	
Low	1 (20)	19 (38.8)	
High	1 (20)	11 (22.4)	
Mineralization lag time, days, median (IQR)	5.9 (5.2–5.9)	21 (13.6–35.6)	0.121
Mineralization, n (%)			1.000
Normal	5 (100)	48 (98)	
Abnormal	0	1 (2)	
BV/TV, %, median (IQR)	16.1 (11.9–17.2)*	19.0 (16.2–23.5)*	0.057
Bone volume, n (%)			0.579
Normal	3 (60)	24 (49)	
Low	2 (40)	12 (24.5)	
High	0	13 (26.5)	

Abbreviations – IQR: interquartile range; BFR/BS: bone formation rate/bone surface; BV/TV: bone volume/tissue volume.

Low bone volume was more frequent in the fracture group (40% vs 25.5%) but the difference was non-significant (p = 0.579). Bone volume (BV/TV) was lower in the fracture group (16.1 (11.9–17.2) vs 19.0 (16.2–23.5), p = 0.057).

### Fracture Risk Evaluation

Median FRAX index was 3.2% (1.9–5.1) for major fracture risk and 0.9% (0.4–1.9) for hip fracture risk. When including CKD as secondary osteoporosis, median FRAX index was 4.6% (2.7–7.6) for major fracture risk and 1.5% (0.6–3.4) for hip fracture.

Median FRAX index for either major fracture or hip fracture was higher in patients who had a fracture during follow-up, as portrayed in Table 3.

**Table 3:** Fracture risk evaluation by FRAX according to fracture status

	Patients with fractures during follow-up n = 5 (9.3%)	Patients without fractures during follow-up n = 49 (90.7%)	p-Value
FRAX (without BMD), median (IQR)	6.2 (3.9–9.2)*	2.9 (1.0–8.7)*	0.003
Major osteoporotic hip fracture	2.2 (1.0–3.2)*	0.7 (0.1–3.3*	0.020
FRAX (without BMD) considering CKD as secondary osteoporosis, median (IQR)	9 (5.6–13)*	3.7 (1.4–13)*	0.003
Major osteoporotic hip fracture	3.8 (1.8–5.7)*	1.2 (0.1–5.7)*	0.016
Intervention threshold with FRAX (without BMD), n (%)	1 (20)	2 (4.5)	0.281
Intervention threshold with FRAX (without BMD) including CKD as secondary osteoporosis, n (%)	4 (80)*	10 (22.7)*	0.019

Abbreviations – BMD: bone mineral density; IQR: interquartile range; CKD: chronic kidney disease.

High fracture risk calculated by FRAX (defined by the intervention threshold: FRAX major fracture risk ≥11% and/or hip fracture risk ≥3%) was more frequent in the fracture group, but this difference was only statistically significant when considering CKD as secondary osteoporosis (80% vs 22.7%, p = 0.019). Fracture risk measured by FRAX was not associated with bone histology subtypes (Table 4).

**Table 4:** Fracture risk evaluation by FRAX according to bone histology

	Normal bone n = 22 (40,7%)	Adynamic bone disease n = 20 (37%)	Hyperparathyroid bone disease n = 11 (20.4%)	p-Value
FRAX (without BMD), median (IQR)				
Major osteoporotic	3.4 (2.4–5.7))	2.5 (1.9–4.1)	2.3 (1.2–4.5)	0.354
Hip Fracture	1 (0.6–2.2)	0.7 (0.4–1.5)	0.6 (0.1–1.4)	0.450
FRAX (without BMD) considering CKD as secondary osteoporosis, n (%), median (IQR)				
Major osteoporotic	4.9 (3.4–8.5)	3.6 (2.8–6)	3.3 (1.7–6.4)	0.292
Hip fracture	1.7 (1.1–3.9)	1.2 (0.7–2.7)	1.1 (0.2–2.4)	0.317

Abbreviations – BMD: bone mineral density; IQR: interquartile range; CKD: chronic kidney disease.

Table 5 presents the results of ROC curve analysis for FRAX. The AUC for FRAX for major fracture risk without adjustments was 0.886 (95%CI 0.781–0.992) and 0.882 (CI 95% 0.777–0.987) when including CKD as secondary osteoporosis. The sensitivity for the prediction of fractures by FRAX was higher when including CKD as secondary osteoporosis (80 vs 20%) and so was the negative predictive value (97.1% vs 91.3%). This increase in sensitivity was accompanied with a slight decrease in specificity (77.3% vs 95%).

**Table 5:** ROC curve analysis for FRAX for fracture prediction

	FRAX (without BMD) including CKD as secondary osteoporosis	FRAX (without BMD)
AUC for major fracture risk	0.882 (95% CI 0.777–0.987)	0.886 (95% CI 0.781–0.992)
Sensitivity	80% (95% CI 0.372–0.987)	20% (95% CI 0.013–0.628)
Specificity	77.3% (95% CI 0.639–0.879)	95% (95% CI 0.866–0.992)
Positive predictive value	28.6% (95% CI 0.099–0.545)	33.3% (95% CI 0.023–0.839)
Negative predictive value	97.1% (95% CI 0.880–0.998)	91.3% (95% CI 0.809–0.972)

Abbreviations – AUC: area under the curve; BMD: bone mineral density; CKD: chronic kidney disease.

## Discussion

The incidence of fragility fractures in our population of CKD patients was 12/1000 patient-year, with 9.3% of the patients experiencing a fracture. Despite the absence of a control group for direct comparison, studies in the general Portuguese population show that this incidence is higher than usual^16,17^. CKD patients have an increased fracture risk^18^. The DOPPS study evaluated 36,337 patients on hemodialysis in 12 countries and showed that fracture incidence varied across countries, from 12 events/1000 patient-year in Japan to 45/1000 patient-year in Belgium, with a follow-up time of 1.6 years^18^. The incidence in our study was lower compared to several of the countries in the DOPPs study. However, we evaluated a predialysis population, with less than half of the patients progressing to hemodialysis or peritoneal dialysis during follow-up (37%). Moreover, we included a small number of patients, which is a limitations the evaluation of an outcome such as fracture.

All included patients were submitted to a bone biopsy at the beginning of the follow-up, known as the gold-standard for the evaluation of bone volume, turnover, and mineralization^14^. Most patients had normal bone histology, and low-turnover bone disease was the more common subtype of ROD. This is in line with recent studies that report an increased prevalence of adynamic bone disease in the earlier stages of CKD^8,19,20^.

The relevance of bone histology subtype in fracture incidence is not clear, with only a few studies on this relationship^13,21^. Additionally, these studies evaluated bone biopsies from patients in dialysis and there is a paucity of data on bone biopsies in CKD stage 3-4^8^. In our study, ROD subtype was not associated with incidence of fractures. When considering BV alone, there was a tendency for an association between low bone volume and occurrence of fractures. This is in agreement with previous series with bone biopsies. The findings confirm that the association of high fracture risk with PTH levels <100 ng/mL may be driven by other factors that influence PTH levels besides adynamic bone disease alone^13,22^. Indeed, there were no differences in PTH levels between the two groups evaluated. High phosphorus level was associated with fractures and have previously been described as a possible risk factor for fractures not only in CKD patients but also in the general population^23,24^. ALP values, contrary to expectations, did not differ between the two groups. This can be explained by the retrospective nature of the study and also by the fact that we did not measure bone-specific alkaline phosphatase (BSAP), which better reflects bone remodeling and has been shown to correlate with risk fracture^25^.

Only 19 patients had a DXA available, and 5 of these presented osteoporosis according to DXA. None of these patients had a fracture during follow-up. These results highlight the limitation of DXA in evaluating fracture risk in CKD patients. Bone strength is dependent on bone quantity and quality. Loss of bone mass increases the risk of fractures, but this is not sufficient to explain the high incidence of fractures in patients with CKD, in whom bone quality has been shown to deteriorate significantly^12^.

On the other hand, FRAX (without BMD), independently of the inclusion of CKD as secondary osteoporosis, showed an good overall diagnostic accuracy for predicting fractures in predialysis CKD. However, considering CKD as a cause of secondary osteoporosis improved the sensitivity of FRAX in this population. This improvement can be of extreme relevance in a group of patients whose fracture risk is underestimated and significantly higher than in the general population^3^. The predictive ability of FRAX in our population indicates that this is useful tool for fracture risk assessment in CKD predialysis patients^26^.

Our study presents several limitations. Firstly, it is a retrospective study, which limits the amount of information gathered, the evaluation of causal associations, and the extrapolation to the general CKD population. The evaluated population was heterogeneous, with some patients remaining in CKD stage 3-4 throughout the study, others progressing to dialysis, and a few dying during the follow-up. The groups with and without fractures were not balanced. In addition, we included a small number of patients, and therefore the evaluation of fractures is limited, as is the evaluation of predictors and fracture risk. Most patients did not have a DXA, and this imposes a limitation on the results regarding the prevalence of densitometric osteoporosis. In the future, studies evaluating the direct relationship between DXA results and bone biopsies can help defining the real value of DXA in CKD patients in clinical practice. Finally, whether BRBs and bone biopsies, performed at the beginning of the follow-up, reflect the bone remodeling activity at the time of fracture is unknown. Bone remodeling rates change over time, and it is likely that kidney function deteriorated by the time of fracture. Studies that perform bone biopsy at the time of fracture would provide better information on the relationship between bone histology and fractures.

Nonetheless, to our knowledge, this is the first study to describe the possible impact of ROD and TMV parameters in the occurrence of fractures and fracture risk in predialysis CKD patients.

In conclusion, our results suggest that different ROD subtypes may not be associated with the incidence of fractures in predialysis CKD patients. However, when considering bone volume alone (but not BMD assessed by DXA), low BV could be a predictor of fracture occurrence. Prospective studies with a larger sample are needed to assess the clinical implication of bone biopsy in fractures and fracture risk in CKD patients.

## Data Availability

Data of the analysis is available upon reasonable request.
