# Supplementary material for: The impact of bone histomorphometry, bone-related biomarkers, and FRAX® on fractures patients with predialysis CKD
Source: J Bras Nefrol. 2025 Dec 1;47(4):e20240276. doi: 10.1590/2175-8239-JBN-2024-0276en (PMC12671521; doi:10.1590/2175-8239-JBN-2024-0276en)
Supplement: Translation

# Translation: Impacto da histomorfometria óssea, biomarcadores ósseos e FRAX^®^ em fraturas em pacientes com DRC em pré-diálise

**DOI:** 10.1590/2175-8239-JBN-2024-0276pt

**Authors:** Mariana Fernandes Diz Lopes, Bernardo José Cardoso Fernandes, Maria Teresa Almeida e Sousa Martins da Rocha, Maria Lúcia Carvalho Costa, João Miguel Machado Dória Frazão, Ricardo de Morais Pereira

**Affiliations:** 1 Unidade Local de Saúde de São João, Departamento de Reumatologia, Porto, Portugal.; 2 Universidade de Porto, Faculdade de Medicina, RISE-Health, Departamento de Medicina, Porto, Portugal.; 3 Unidade Local de Saúde de São João, Departamento de Nefrologia, Porto, Portugal.

**Keywords:** Doença Renal Crônica – Distúrbio Mineral Ósseo, Fraturas, Fraturas Osteoporóticas, Osteoporose, Histomorfometria, Remodelação Óssea, Banco de Ossos

## Abstract

**Introdução:**

Pacientes com doença renal crônica (DRC) apresentam risco aumentado de fraturas. Atualmente, não está estabelecido se o tipo de osteodistrofia renal (ODR) contribui para esse risco, pois biópsias ósseas não são frequentemente realizadas na prática clínica. Nosso objetivo foi avaliar a associação entre subtipos de ODR, biomarcadores ósseos e risco de fraturas determinado pela ferramenta FRAX^®^ com a ocorrência de fraturas em pacientes com DRC pré-diálise.

**Métodos:**

Estudo retrospectivo com pacientes acompanhados entre 2014–2023. Realizaram-se exames de sangue – incluindo biomarcadores ósseos (BRB) – e biópsias ósseas no início do acompanhamento. Dados de densitometria óssea (DXA) e fraturas clinicamente evidentes foram obtidos de registros médicos. Avaliaram-se radiografias da coluna torácica/lombar para detectar fraturas vertebrais. O índice FRAX^®^, sem densidade mineral óssea (DMO), foi calculado com ferramenta online.

**Resultados:**

O tempo médio de acompanhamento foi 7,5 ± 3 anos; 9,3% dos pacientes apresentaram fratura óssea, com incidência de 12/1000 pacientes-ano. Pacientes que sofreram fratura exibiram níveis mais elevados de fósforo (4,1 mg/dL vs. 3,5 mg/dL; p = 0,047). Os subtipos histomorfométricos e os BRB não foram associados à incidência de fraturas nem ao risco de fraturas avaliado pelo FRAX^®^. Houve tendência a menor volume ósseo no grupo com fraturas (p = 0,057). FRAX^®^ (sem DMO), independen­temente da inclusão da DRC como osteoporose secundária, apresentou boa precisão diagnóstica global para prever fraturas na DRC pré-diálise.

**Conclusão:**

Os subtipos de ODR não foram associados à incidência de fraturas nesses pacientes. A capacidade discriminatória do FRAX nessa população enfatiza sua utilidade em pacientes com DRC pré-diálise.

## Introdução

A osteoporose é um distúrbio esquelético comum que afeta a macro e microarquitetura óssea, bem como a densidade mineral óssea (DMO), resultando em fragilidade óssea e fraturas por fragilidade^1^. Todas as fraturas representam um grande ônus, uma vez que estão associadas à elevada morbimortalidade^1^. Pacientes com doença renal crônica (DRC) apresentam uma fisiopatologia complexa de fragilidade óssea devido ao desenvolvimento de distúrbios minerais ósseos (DMO-DRC), apresentando risco aumentado de fraturas^2^. A DMO-DRC inclui distúrbios metabólicos do cálcio, fósforo, paratormônio e fator de crescimento de fibroblastos (FGF)-23 que levam à alteração da morfologia óssea (osteodistrofia renal), calcificação vascular e morte cardiovascular^3^. A classificação TMV (*turnover* [remodelação], *mineralization* [mineralização] e *bone volume* [volume ósseo]), proposta pelo *Kidney Disease: Improving Global Outcomes* (KDIGO), é utilizada para estabelecer os diferentes subtipos de osteodistrofia renal (ODR), a qual é avaliada por meio de biópsias ósseas e análise histomorfométrica^4^.

Em pacientes com DRC estágios 1-3, o risco de osteoporose e fraturas é semelhante ao da população em geral^5,6^. No entanto, surgem desafios ao lidar com pacientes com DRC nos estágios 4-5^5,6^. A densitometria óssea (DXA) é considerada o padrão-ouro para a avaliação da DMO e do risco de fraturas na prática clínica^7^. No entanto, a DXA não distingue entre osso cortical e trabecular, não diferencia entre condições de volume ósseo e mineralização óssea e pode ser influenciada por artefatos, como calcificações ectópicas ou formação óssea patológica^2,5,6^. Portanto, a precisão do teste pode ser comprometida em algumas populações específicas, como pacientes com DRC.

O valor dos biomarcadores ósseos circulantes no risco de fraturas não está estabelecido^8^. Diversas moléculas são relevantes no metabolismo ósseo e mineral. O paratormônio (PTH) desempenha um papel central na remodelação óssea e na via Wnt/beta-catenina; a fosfatase alcalina óssea (BALP) e a fosfatase alcalina total (ALP) são marcadores da atividade de formação óssea; o fator de crescimento de fibroblastos 23 (FGF-23) é um hormônio pleiotrópico que inibe a reabsorção de fosfato e a produção de calcitriol; a esclerostina e a proteína relacionada ao Dickkopf 1 (Dkk-1) são antagonistas da osteoblastogênese e afetam indiretamente o ligante do receptor ativador do fator nuclear kB (RANKL), que se liga ao receptor RANK nos osteoclastos e estimula a reabsorção óssea e a osteoprotegerina, bloqueando a interação entre RANK e RANKL^9^.

A Avaliação do Risco de Fratura (FRAX^®^) é uma ferramenta online disponível mundialmente, utilizada para predizer o risco de fratura em 10 anos^10^. Embora seja empregada em pacientes com DRC, ela pode subestimar o risco de fratura. Portanto, alguns ajustes são necessários para aprimorar sua precisão^11^.

Prever o risco de fraturas em pacientes com DRC ainda representa um desafio na prática clínica. A resistência óssea é determinada não apenas pela quantidade de massa óssea (avaliada principalmente pela DMO, por meio da DXA), mas também pela qualidade óssea, determinada pela microarquitetura e pelas propriedades mecânicas do osso^12^. Atualmente, não se sabe se os subtipos de ODR e os parâmetros de classificação TMV (remodelação, mineralização e volume) contribuem para a avaliação da ocorrência e do risco de fraturas. Um estudo de grande escala, realizado no Brasil e no Uruguai, não foi capaz de encontrar uma associação entre ODR e a incidência de fraturas^5,13^. Como as biópsias ósseas não são realizadas rotineiramente devido ao seu caráter invasivo e à necessidade de equipamentos e treinamento específicos para a análise histomorfométrica^14^, o uso de ferramentas não invasivas, como o FRAX, deve ser ampliado.

Os objetivos deste estudo foram: (i) avaliar a incidência de fraturas em uma coorte de pacientes com DRC em pré-diálise; (ii) estudar a associação entre os subtipos de ODR e biomarcadores ósseos circulantes com fraturas; e (iii) avaliar o valor preditivo do índice FRAX para fraturas osteoporóticas maiores nessa população.

## Métodos

### Desenho e População do Estudo

Este estudo foi uma avaliação retrospectiva de pacientes adultos acompanhados em nossa instituição entre 2014 e 2023, submetidos a biópsia óssea durante o período pré-diálise, previamente incluídos em um estudo transversal realizado por Pereira et al^8^. Os pacientes eram elegíveis se apresentassem DRC em estágio 3-4 no momento da biópsia óssea, fossem capazes de fornecer consentimento informado e tivessem mais de 18 anos de idade. Os critérios de exclusão foram histórico de tratamento com bisfosfonatos, denosumabe ou osteoanabólicos (teriparatida), além de paratireoidectomia prévia. Todos os pacientes assinaram um termo de consentimento informado. O protocolo do estudo foi aprovado pelo Comitê de Ética Institucional.

### Exames Laboratoriais

Todos os pacientes tiveram amostras de sangue coletadas no momento da biópsia óssea. Cálcio, fósforo, albumina, creatinina e fosfatase alcalina séricos foram mensurados utilizando um analisador Olympus AU5400 (*Olympus America*, *Centre Valley*, PA, EUA). Os níveis séricos de iPTH e 25-hidroxivitamina D [25(OH)D] foram avaliados por meio de imunoensaio por eletroquimioluminescência em um analisador Cobas E411 (*Roche Diagnostics*, Mannheim, Alemanha). A esclerostina bioativa e o RANKL solúvel livre (sRANKL) foram medidos em amostras de plasma por meio de imunoensaio enzimático (*Biomedica Medizinprodukte GmbH*, Viena, Áustria), de acordo com as instruções do fabricante (faixa de detecção de 1,9–320 pmol/L e 0,2–40 pg/mL, respectivamente). A osteoprotegerina, o DKK1 e o FGF-23 intacto foram mensurados em amostras de plasma por meio de um ensaio multiplex (*Magnetic Luminex Assay*, *R&D Systems Inc.*, Minneapolis, MN, EUA), de acordo com o protocolo do fabricante (faixa de detecção de 75–18.220 pg/mL, 202–49.060 pg/mL e 11,8–2870 pg/mL, respectivamente). O estadiamento da DRC foi definido de acordo com a equação da *CKD Epidemiology Collaboration*.

### Biópsia Óssea e Histomorfometria

Todos os pacientes foram submetidos a uma biópsia óssea transilíaca com um trépano de Bordier modificado no início do acompanhamento. A biópsia óssea foi realizada após um ciclo de dupla marcação com tetraciclina (doxiciclina 200 mg, duas vezes ao dia, durante 3 dias, repetido após 12 dias; a biópsia foi realizada 3 dias após a segunda marcação). Os fragmentos de biópsia apresentavam 5 mm de diâmetro e 10 mm de comprimento. O material foi desidratado em álcool, diafanizado em xilol e embebido em metacrilato de metila. Secções de 5 μm não descalcificadas foram cortadas e coradas com tricrômico de Masson-Goldner modificado para avaliação histomorfométrica estática. Foram preparadas secções de 10 μm não coradas para análise por microscopia de fluorescência dos parâmetros dinâmicos. A histomorfometria óssea foi realizada com o software *OsteoMeasure* (*OsteoMetrics*, *Decatur*, GA, EUA). Os parâmetros estáticos e dinâmicos foram analisados de acordo com as normas estabelecidas pela *American Society of Bone and Mineral Research* ^15^.

Seguindo as diretrizes do KDIGO^4^, a ODR foi categorizada de acordo com a classificação TMV em: osteomalácia (baixa remodelação, mineralização anormal), osso adinâmico (baixa remodelação, mineralização normal), osteodistrofia urêmica mista (alta remodelação, mineralização anormal) e doença óssea relacionada ao hiperparatireoidismo (alta remodelação, mineralização normal).

A baixa remodelação óssea foi definida como taxa de formação óssea/superfície óssea (BFR/BS) <10,95 mm^3^/mm^2^/ano e a alta remodelação óssea como BFR/BS >37,5 mm^3^/mm^2^/ano. O volume ósseo foi considerado normal se volume ósseo/volume tecidual (BV/TV) >16%. A mineralização foi considerada normal se o intervalo de tempo para mineralização (Mlt) fosse <50 dias. As biópsias foram avaliadas por dois observadores diferentes.

### Fraturas e Avaliação do Risco de Fraturas

Uma fratura por fragilidade foi definida como aquela resultante de um trauma equivalente ou inferior a uma queda da própria altura. Foram incluídas fraturas vertebrais e não vertebrais por fragilidade. Os registros médicos foram avaliados quanto à ocorrência de fraturas clínicas (sintomáticas). Radiografias laterais da coluna vertebral, realizadas nos últimos dois anos, estavam disponíveis para 51 pacientes e foram revisadas por um reumatologista para a detecção de fraturas vertebrais assintomáticas, utilizando o método semiquantitativo de Genant.

Todas as informações sobre fatores de risco para fraturas ósseas foram obtidas a partir do histórico médico, incluindo idade, sexo, peso e altura, fraturas anteriores, histórico familiar de fraturas de quadril, tabagismo, consumo de álcool (>3 U/dia), artrite reumatoide, uso atual ou prévio de corticosteroides sistêmicos por >3 meses em dose >5 mg/dia e diagnóstico de doença que induz osteoporose secundária (doença hepática crônica, diabetes mellitus tipo 1, menopausa precoce [<45 anos], osteogênese imperfeita, hipertireoidismo não tratado, hipogonadismo).

O risco de fratura óssea maior e de quadril em 10 anos foi calculado com a versão em português da calculadora FRAX (https://frax.shef.ac.uk/FRAX/tool.aspx?country=53). O risco elevado de fraturas segundo o índice FRAX sem DMO, também considerado como o ponto de corte para intervenção terapêutica, foi definido como ≥11% para fraturas maiores e ≥3% para fraturas da anca^7^.

Os dados de DXA foram coletados quando disponíveis. A DMO do colo do fêmur, quadril total e coluna lombar (L1–L4) foi avaliada com o equipamento *QDR Hologic Delphi*.

### Análise Estatística

A análise estatística foi realizada utilizando o SPSS versão 29.0 (IBM SPSS *Statistics*, Chicago, IL, EUA). Os dados contínuos foram expressos como média e desvio padrão (DP) ou mediana e intervalo interquartil (IIQ), de acordo com a distribuição das variáveis. As variáveis categóricas foram expressas como frequências e porcentagens.

Foram comparados os grupos com e sem fraturas, e os valores de p foram calculados por meio dos testes de Mann-Whitney U e Kruskal Wallis para variáveis contínuas e dos testes qui-quadrado e exato de Fisher para variáveis categóricas.

Para determinar a capacidade da ferramenta FRAX em predizer fraturas, foram construídas curvas ROC (*receiver operating characteristic*) para cada variável preditiva: FRAX sem DMO e FRAX sem DMO considerando DRC como osteoporose secundária. Avaliamos as áreas sob a curva ROC (AUC) para cada modelo FRAX. Uma AUC foi considerada boa se >0,7 e excelente se >0,8. Foram calculados a sensibilidade, a especificidade e os valores preditivos positivos e negativos. Valores de p <0,05 foram considerados estatisticamente significativos.

## Resultados

### Características Clínicas, Demográficas e Laboratoriais de Acordo com o *Status* da Fratura

Foram incluídos 54 pacientes, a maioria do sexo masculino (n = 43; 79,6%) e com idade média de 72,2 ± 10,2 anos. O tempo mediano de acom­panhamento foi de 7,5 ± 3 anos. Dois pacientes (3,7%) apresentaram fraturas clinicamente evidentes (uma fratura de quadril e uma fratura do osso peroneal) e foram identificadas fraturas vertebrais assintomáticas nas radiografias de 3 (5,9%) pacientes. Isso representa uma incidência de fraturas de 12/1000 pacientes-ano, com 9,3% dos pacientes apresentando alguma fratura.

As características clínicas, demográficas e bioquímicas dos dois grupos estão resumidas na Tabela 1. A idade média no momento da biópsia óssea foi de 71,84 ± 4,4 anos, no grupo com fratura, e 64,7 ± 10 anos, no grupo sem fratura. A maioria dos pacientes era do sexo masculino em ambos os grupos (n = 3; 60% e n = 40; 81,6%).

**Tabela 1:** Características clínicas, demográficas e laboratoriais de acordo com o status da fratura

	Pacientes com fraturas durante o acompanhamento n = 5 (9,3%)	Pacientes sem fraturas durante o acompanhamento n = 49 (90,7%)	Valor de p
Idade (anos) no momento da biópsia óssea, média (DP)	71,8 (4,4)	64,7 (10)	0,126
Idade (anos) ao final do acompanhamento, média (DP)	79 (5,4)	71,5 (10,4)	0,117
Estágio da DRC, n (%)			0,358
Estágio 3	1 (20)	24 (49)	
Estágio 4	4 (80)	25 (51)	
Sexo			0,266
Feminino, n (%)	2 (40)	9 (18,4)	
Masculino, n (%)	3 (60)	40 (81,6)	
Etiologia da doença renal crônica, n (%)			0,732
Hipertensão	3 (60)	13 (26,5)	
Diabetes mellitus	2 (40)	13 (26,5)	
Glomerulonefrite crônica	0	6 (12,2)	
Nefrite tubulointersticial crônica	0	8 (16,3)	
Doença renal policística autossômica dominante	0	2 (4,1)	
Isquêmica	0	1 (2)	
Indeterminada	0	6 (12,2)	
Fumante atual, n (%)	0	1 (1,9)	0,747
Consumo de álcool, n (%)	0	4 (8,2)	0,670
Peso, mediana (IIQ)	79 (16)	76 (16,5)	0,900
Altura, média (DP)	158,4 (6,7)	163 (7,9)	0,215
Índice de massa corporal, mediana (IIQ)	32,8 (8,4)	28,1 (5,4)	0,366
Bioquímica, mediana (IIQ)			
Creatinina, mg/dL	2,2 (1,1)	2,2 (0,5)	0,415
TFG, mL/min/1,73 m^2^	26,5 (15)	30,5 (12)	0,157
Hemoglobina, g/dL	11,5 (0,5)*	13,3 (1,9)*	0,008
Albumina, g/dL	4,08 (0,14)	4,2 (0,4)	0,848
Cálcio, mg/dL	4,7 (0)	4,8 (0,4)	0,685
Fósforo, mg/dL	4,1 (0,8)*	3,5 (0,9)*	0,047
Vitamina D 25(OH), ng/mL	12,5 (4,8)	16 (12,3)	0,221
Magnésio, mEq/L	1,8 (0,3)	1,6 (0,3)	0,957
Proteína C reativa, mg/L	3,9 (4,4)	1,5 (3,5)	0,662
25(OH)D, ng/mL	13,5 (3)	16 (9,8)	0,221
ALP, U/L	75,5 (19)	71 (43)	0,815
iPTH, pg/mL	105,7 (40,6)	79,9 (93,7)	0,728
FGF-23, pg/mL	30,9 (10,8)	22,1 (21,1)	0,874
Esclerostina, pmol/L	83,9 (105)	58,4 (44,7)	0,986
DKK1, pg/mL	1004,5 (283,9)	738 (498,9)	0,366
sRANKL, pg/mL	2,7 (0,3)	2,5 (1,4)	0,231
Osteoprotegerina, pg/mL	1554,4 (408,3)	1404,1 (545,3)	0,385
Osteoporose segundo DXA, n (%)	0 (0)	5 (9,3)	0,665
Progressão para terapia renal substitutiva durante o acompanhamento, n (%)			1,000
Hemodiálise	2 (40)	18 (36,7)	
Diálise peritoneal	0	2 (4)	
Óbito durante o acompanhamento, n (%)	1 (20)	4 (8,2)	0,397

Abreviaturas – DP: desvio padrão; DRC: doença renal crônica; IIQ: intervalo interquartil; TFG: taxa de filtração glomerular; ALP: fosfatase alcalina; iPTH: paratormônio intacto; FGF-23: fator de crescimento de fibroblastos 23; DKK1: proteína relacionada ao Dickkopf 1; RANKL: ligante do receptor ativador do fator nuclear kB solúvel.

Hipertensão (n = 3; 60%) e diabetes (n = 2; 40%) foram as etiologias da DRC em todos os pacientes do grupo com fratura. A maioria dos pacientes apresentou DRC estágio 4 (n = 29; 53,7%) e, durante o acompanhamento, 20 pacientes evoluíram para hemodiálise, 2 para diálise peritoneal e 5 foram a óbito (9,3%). Não houve diferenças estatisticamente significativas entre os dois grupos quanto à progressão dos pacientes para terapia renal substitutiva.

Cinco pacientes (9,3%) apresentaram osteoporose de acordo com o escore T da DXA e nenhum deles sofreu fratura durante o acompanhamento.

A média dos níveis séricos de cálcio e fósforo no momento da biópsia óssea foi de 9,4 ± 0,7 mg/dL e 3,5 ± 0,7 mg/dL, respectivamente. A mediana dos níveis séricos de iPTH foi de 90,2 (85,4) pg/mL. Os pacientes que sofreram fraturas apresentaram níveis mais elevados de fósforo (4,1 mg/dL vs. 3,5 mg/dL; p = 0,047) e níveis mais baixos de hemoglobina (11,5 mg/dL vs. 13,3 mg/dL; p = 0,008).

Houve uma tendência para níveis mais elevados de ALP e iPTH no grupo com fraturas, mas essas diferenças não foram significativas. Os demais biomarcadores ósseos circulantes (FGF-23, esclerostina, DKK1, sRANKL e osteoprotegerina) não foram associados à incidência de fraturas.

### Análise da Histomorfometria Óssea e Fraturas

Na análise histomorfométrica, 40,7% (n = 22) dos pacientes apresentaram histologia óssea normal, 37% (n = 20) apresentaram baixa remodelação óssea com mineralização normal (doença óssea adinâmica), 20,4% (n = 11) apresentaram alta remodelação óssea com mineralização normal (doença óssea associada ao hiperparatireoidismo) e 1 paciente apresentou osteodistrofia urêmica mista (alta remodelação óssea com mineralização anormal). Não foram detectados casos de baixa remodelação óssea com mineralização anormal (osteomalácia). As medidas histomorfométricas e a histologia óssea em relação à ocorrência de fraturas são descritas na Tabela 2. Não houve associação entre os subtipos histomorfométricos e a ocorrência de fraturas.

**Tabela 2:** Histologia óssea e parâmetros histomorfométricos de acordo com o status da fratura

	Pacientes com fraturas durante o acompanhamento n = 5 (9,3%)	Pacientes sem fraturas durante o acompanhamento n = 49 (90,7%)	Valor de p
Resultados de histomorfometria óssea, n (%)			0,847
Osso normal	3 (60)	19 (38,8)	
Doença óssea adinâmica	1 (20)	19 (38,8)	
Doença óssea por hiperparatireoidismo	1 (20)	10 (20,4)	
Osteodistrofia urêmica mista	0 (0)	1 (2)	
BFR/BS, mm3/mm2/ano, mediana (IIQ)	15,5 (5,2–20,4)	15,5 (8,9–38,0)	0,303
Remodelação, n (%)			0,833
Normal	3 (60)	19 (38,8)	
Baixa	1 (20)	19 (38,8)	
Alta	1 (20)	11 (22,4)	
Intervalo de tempo para mineralização, dias, mediana (IIQ)	5,9 (5,2–5,9)	21 (13,6–35,6)	0,121
Mineralização, n (%)			1,000
Normal	5 (100)	48 (98)	
Anormal	0	1 (2)	
BV/TV, %, mediana (IIQ)	16,1 (11,9–17,2)*	19,0 (16,2–23,5)*	0,057
Volume ósseo, n (%)			0,579
Normal	3 (60)	24 (49)	
Baixo	2 (40)	12 (24,5)	
Alto	0	13 (26,5)	

Abreviaturas – IIQ: intervalo interquartil; BFR/BS: taxa de formação óssea/superfície óssea; BV/TV: volume ósseo/volume tecidual.

O baixo volume ósseo foi mais frequente no grupo com fraturas (40% vs. 25,5%), mas a diferença não foi significativa (p = 0,579). O volume ósseo (BV/TV) foi menor no grupo com fraturas (16,1 (11,9–17,2) vs. 19,0 (16,2–23,5); p = 0,057).

### Avaliação do Risco de Fraturas

A mediana do índice FRAX foi de 3,2% (1,9–5,1) para o risco de fraturas maiores e de 0,9% (0,4–1,9) para o risco de fraturas de quadril. Ao incluir a DRC como osteoporose secundária, o índice FRAX mediano foi de 4,6% (2,7–7,6) para o risco de fraturas maiores e 1,5% (0,6–3,4) para fraturas de quadril.

O índice FRAX mediano tanto para fraturas maiores quanto para as de quadril foi mais elevado em pacientes que sofreram fraturas durante o acompanhamento, conforme ilustrado na Tabela 3.

**Tabela 3:** Avaliação do risco de fratura pelo FRAX de acordo com o status da fratura

	Pacientes com fraturas durante o acompanhamento n = 5 (9,3%)	Pacientes sem fraturas durante o acompanhamento n = 49 (90,7%)	Valor de p
FRAX (sem DMO), mediana (IIQ)	6,2 (3,9–9,2)*	2,9 (1,0–8,7)*	0,003
Fratura osteoporótica maior/de quadril	2,2 (1,0–3,2)*	0,7 (0,1–3,3)*	0,020
FRAX (sem DMO) considerando DRC como osteoporose secundária, mediana (IIQ)	9 (5,6–13)*	3,7 (1,4–13)*	0,003
Fratura osteoporótica maior/de quadril	3,8 (1,8–5,7)*	1,2 (0,1–5,7)*	0,016
Limiar de intervenção com FRAX (sem DMO), n (%)	1 (20)	2 (4,5)	0,281
Limiar de intervenção com FRAX (sem DMO) incluindo DRC como osteoporose secundária, n (%)	4 (80)*	10 (22,7)*	0,019

Abreviaturas – DMO: densidade mineral óssea; IIQ: intervalo interquartil; DRC: doença renal crônica.

O risco elevado de fratura calculado pelo FRAX (definido pelo limiar de intervenção: risco FRAX de fratura maior ≥11% e/ou de fratura de quadril ≥3%) foi mais frequente no grupo com fratura, mas essa diferença só foi estatisticamente significativa quando se considerou a DRC como osteoporose secundária (80% vs. 22,7%; p = 0,019). O risco de fratura mensurado pelo FRAX não esteve associado aos subtipos histológicos ósseos (Tabela 4).

**Tabela 4:** Avaliação do risco de fratura pelo FRAX de acordo com a histologia óssea

	Ossos normais n = 22 (40,7%)	Doença óssea adinâmica n = 20 (37%)	Doença óssea por hiperparatireoidismo n = 11 (20,4%)	Valor de p
FRAX (sem DMO), mediana (IIQ)	3,4 (2,4–5,7)	2,5 (1,9–4,1)	2,3 (1,2–4,5)	0,354
Fratura osteoporótica maior/de quadril	1 (0,6–2,2)	0,7 (0,4–1,5)	0,6 (0,1–1,4)	0,450
FRAX (sem DMO) considerando a DRC como osteoporose secundária, n (%), mediana (IIQ)	4,9 (3,4–8,5)	3,6 (2,8–6)	3,3 (1,7–6,4)	0,292
Fratura osteoporótica maior/de quadril	1,7 (1,1–3,9)	1,2 (0,7–2,7)	1,1 (0,2–2,4)	0,317

Abreviaturas – DMO: densidade mineral óssea; IIQ: intervalo interquartil; DRC: doença renal crônica.

A Tabela 5 apresenta os resultados da análise da curva ROC para o FRAX. A AUC para o FRAX referente ao risco de fraturas maiores sem ajustes foi de 0,886 (IC95% 0,781–0,992), e 0,882 (IC95% 0,777–0,987) quando considerada a DRC como osteoporose secundária. A sensibilidade para a predição de fraturas pelo FRAX foi maior quando se incluiu a DRC como osteoporose secundária (80% vs. 20%), assim como o valor preditivo negativo (97,1% vs. 91,3%). Esse aumento na sensibilidade foi acompanhado por uma ligeira redução na especificidade (77,3% vs. 95%).

**Tabela 5:** Análise da curva ROC do FRAX para predição de fraturas

	FRAX (sem DMO) incluindo DRC como osteoporose secundária	FRAX (sem DMO)
AUC para risco de fratura maior	0.882 (95% CI 0.777–0.987)	0.886 (95% CI 0.781–0.992)
Sensibilidade	80% (95% CI 0.372–0.987)	20% (95% CI 0.013–0.628)
Especificidade	77.3% (95% CI 0.639–0.879)	95% (95% CI 0.866–0.992)
Valor preditivo positivo	28.6% (95% CI 0.099–0.545)	33.3% (95% CI 0.023–0.839)
Valor preditivo negativo	97.1% (95% CI 0.880–0.998)	91.3% (95% CI 0.809–0.972)

Abreviaturas – AUC: área sob a curva; DMO: densidade mineral óssea; DRC: doença renal crônica.

## Discussão

A incidência de fraturas por fragilidade em nossa população de pacientes com DRC foi de 12/1000 pacientes-ano, com 9,3% dos pacientes apresentando alguma fratura. Apesar da ausência de um grupo controle para comparação direta, estudos na população geral portuguesa demonstram que essa incidência é superior ao habitual^16,17^. Pacientes com DRC apresentam um risco aumentado de fraturas^18^. O estudo DOPPS avaliou 36.337 pacientes em hemodiálise em 12 países e demonstrou que a incidência de fraturas variou entre eles, de 12 eventos/1000 pacientes-ano no Japão a 45/1000 pacientes-ano na Bélgica, com tempo de acompanhamento de 1,6 anos^18^. A incidência em nosso estudo foi menor em comparação a vários dos países do estudo DOPPs. No entanto, avaliamos uma população em pré-diálise, com menos da metade dos pacientes progredindo para hemodiálise ou diálise peritoneal durante o acompanhamento (37%). Além disso, incluímos um pequeno número de pacientes, o que representa uma limitação na avaliação de um desfecho como a fratura.

Todos os pacientes incluídos foram submetidos a uma biópsia óssea no início do acompanhamento, considerada o padrão-ouro para a avaliação do volume ósseo, da remodelação e da mineralização^14^. A maioria dos pacientes apresentou histologia óssea normal, e a doença óssea de baixa remodelação foi o subtipo mais comum de ODR. Esse achado está em consonância com estudos recentes que relatam um aumento da prevalência de doença óssea adinâmica nos estágios iniciais da DRC^8,19,20^.

A relevância do subtipo da histologia óssea na incidência de fraturas não é clara, havendo somente alguns estudos sobre essa relação^13,21^. Além disso, esses estudos avaliaram biópsias ósseas de pacientes em diálise, e há escassez de dados sobre tais procedimentos em pacientes com DRC nos estágios 3-4^8^. Em nosso estudo, o subtipo de ODR não esteve associado à incidência de fraturas. Ao considerar o volume ósseo (BV) isoladamente, observou-se uma tendência de associação entre baixo BV e ocorrência de fraturas. Isso está de acordo com séries anteriores envolvendo biópsias ósseas. Os achados confirmam que a associação entre o risco elevado de fraturas e níveis de PTH <100 ng/mL pode ser motivada por outros fatores que influenciam os níveis de PTH, além da doença óssea adinâmica isolada^13,22^. De fato, não foram observadas diferenças nos níveis de PTH entre os dois grupos avaliados. Níveis elevados de fósforo foram associados a fraturas e já foram descritos anteriormente como um possível fator de risco para fraturas, não apenas em pacientes com DRC, mas também na população em geral^23,24^. Contrariamente ao esperado, os valores de ALP não diferiram entre os dois grupos. Esse fato pode ser explicado pela natureza retrospectiva do estudo e também pelo fato de não termos mensurado a fosfatase alcalina óssea específica (BSAP), que reflete de forma mais precisa a remodelação óssea e tem demonstrado correlação com o risco de fraturas^25^.

Somente 19 pacientes dispunham de DXA, e 5 deles apresentaram osteoporose de acordo com o exame. Nenhum desses pacientes apresentou fratura durante o acompanhamento. Esses resultados ressaltam a limitação da DXA na avaliação do risco de fratura em pacientes com DRC. A resistência óssea depende tanto da quantidade de massa óssea quanto da qualidade do osso. A perda de massa óssea aumenta o risco de fraturas, mas tal fato não é suficiente para explicar a alta incidência de fraturas em pacientes com DRC, nos quais a qualidade óssea demonstrou deteriorar-se de modo significativo^12^.

Por outro lado, o FRAX (sem DMO), independentemente da inclusão da DRC como osteoporose secundária, apresentou boa precisão diagnóstica global para predizer fraturas em pacientes com DRC pré-diálise. No entanto, considerar a DRC como causa de osteoporose secundária melhorou a sensibilidade do FRAX nessa população. Esta melhora pode ser de extrema relevância em um grupo de pacientes cujo risco de fratura é subestimado e significativamente maior do que na população em geral^3^. A capacidade preditiva do FRAX em nossa população indica que se trata de uma ferramenta útil para a avaliação do risco de fratura em pacientes com DRC em pré-diálise^26^.

Nosso estudo apresenta diversas limitações. Em primeiro lugar, trata-se de um estudo retrospectivo, o que limita a quantidade de informações coletadas, a avaliação de associações causais e a extrapolação para a população geral com DRC. A população avaliada apresentou heterogeneidade, com alguns pacientes permanecendo no estágio 3-4 da DRC durante todo o estudo, outros progredindo para diálise e alguns indo a óbito durante o acompanhamento. Os grupos com e sem fraturas não apresentaram equilíbrio. Além disso, incluímos um número reduzido de pacientes e, portanto, a avaliação de fraturas é limitada, assim como a análise de preditores e do risco de fraturas. A maioria dos pacientes não realizou DXA, o que impõe uma limitação aos resultados referentes à prevalência de osteoporose densitométrica. No futuro, estudos que avaliem a relação direta entre os resultados da DXA e as biópsias ósseas poderão ajudar a definir o valor real da DXA em pacientes com DRC na prática clínica. Por fim, não se sabe se os BRBs e as biópsias ósseas, realizados no início do acompanhamento, refletem a atividade de remodelação óssea no momento da fratura. As taxas de remodelação óssea se alteram ao longo do tempo, e é provável que a função renal estivesse deteriorada no momento da fratura. Estudos que realizam biópsia óssea no momento da fratura forneceriam informações mais precisas sobre a relação entre a histologia óssea e as fraturas.

No entanto, até onde temos conhecimento, este é o primeiro estudo a descrever o possível impacto da ODR e dos parâmetros TMV na ocorrência de fraturas e no risco de fraturas em pacientes com DRC em pré-diálise.

Em conclusão, nossos resultados sugerem que diferentes subtipos de ODR podem não estar associados à incidência de fraturas em pacientes com DRC pré-diálise. No entanto, quando se considera o volume ósseo isoladamente (mas não a DMO avaliada por DXA), o BV reduzido pode ser um preditor da ocorrência de fraturas. São necessários estudos prospectivos com uma amostra maior para avaliar a implicação clínica da biópsia óssea em fraturas e no risco de fraturas em pacientes com DRC.

## Data Availability

Todo o conjunto de dados que dá suporte aos resultados deste estudo está disponível mediante solicitação ao autor correspondente (Mariana Diz-Lopes).
